# Silica Encapsulation of Hydrophobic Optical NP-Embedded Silica Particles with Trimethoxy(2-Phenylethyl)silane

**DOI:** 10.3390/nano13142145

**Published:** 2023-07-24

**Authors:** Eunil Hahm, Ahla Jo, Eunji Kang, Kwanghee Yoo, Minsup Shin, Jaehyun An, Xuanhung Pham, Hyungmo Kim, Homan Kang, Jaehi Kim, Bonghyun Jun

**Affiliations:** 1 Department of Bioscience and Biotechnology, Konkuk University, Seoul 05029, Republic of Koreadnjzj159159@konkuk.ac.kr (M.S.); wogus4067@naver.com (J.A.);; 2 Gordon Center for Medical Imaging, Department of Radiology, Massachusetts General Hospital, Harvard Medical School, Boston, MA 02114, USA

**Keywords:** silica encapsulation, silica-encapsulated nanoparticles, trimethoxy(2-phenylethyl)silane

## Abstract

Nanoparticles (NP) with optical properties embedded silica particles have been widely used in various fields because of their unique properties. The surfaces of optical NPs have been modified with various organic ligands to maintain their unique optical properties and colloidal stability. Among the surface modification methods, silica encapsulation of optical NPs is widely used to enhance their biocompatibility and stability. However, in the case of NPs with hydrophobic ligands on the surface, the ligands that determine the optical properties of the NPs may detach from the NPs, thereby changing the optical properties during silica encapsulation. Herein, we report a generally applicable silica encapsulation method using trimethoxy(2-phenylethyl)silane (TMPS) for non-hydrophilic optical NPs, such as quantum dots (QDs) and gold NPs. This silica encapsulation method was applied to fabricate multiple silica-encapsulated QD-embedded silica NPs (SiO_2_@QD@SiO_2_ NPs; QD^2^) and multiple silica-encapsulated gold NP-embedded silica NPs labeled with 2-naphthalene thiol (SiO_2_@Au_2-NT_@SiO_2_). The fabricated silica-encapsulated NPs exhibited optical properties without significant changes in the quantum yield or Raman signal intensity.

## 1. Introduction

Nanoparticles (NPs) with optical properties, such as plasmonic NPs and fluorescent quantum dots (QDs), have been extensively utilized in various fields, including biomedical imaging, drug delivery, sensing, catalysis, and optoelectronics [1,2,3,4]. To improve the biocompatibility of NPs and impart them with additional merits, researchers have focused on a silica encapsulation method for optical NPs [5,6,7,8,9]. Silica-encapsulated NPs have several advantages over organic ligand-coated NPs. First, the outer silica shell prevents direct contact between the encapsulated NPs and the external environment; thus, the silica-encapsulated NPs remain unaffected by degradation or oxidation and do not exhibit cytotoxicity [10,11]. Second, the silica shell enhances the hydrophilicity of the NPs and prevents their aggregation under physiological conditions. Unlike commonly used hydrophobic organic ligands, such as oleic acid or trioctylphosphine oxide, silica-encapsulated NPs are hydrophilic with silanol groups in the silica shell. Finally, the silica shell provides flexibility for surface modification. Silanol groups can easily react with various silanol precursors with functional groups, such as amine or thiol groups; therefore, various functional groups like amine or thiol groups can be introduced with other silylether-containing compounds, such as 3-aminopropyl triethoxysilane (APTES), 3-chloropropyl trimethoxysilane (CPTMS), 3-glycydoxypropyl trimethoxysilane (GPTS), or 3-mercaptopropyl trimethoxysilane (MPTS) [12,13,14,15].

Embedding optical NPs into silica particles has significant potential in bio applications as it enables enhanced and/or tunable optical properties and ease of handling. Focusing on these merits, we have reported various types of silica-encapsulated NPs, including silica templates and surface modification methods using MPTS and tetraethyl orthosilicate (TEOS) [16,17,18,19,20,21]. Because the thiol group of MPTS has a high affinity for optical NPs, such as Au or QDs, silica precursors can be easily introduced on the surface of the optical NPs, and easily forming a silica shell. Their unique optical properties are retained after silica shell coating, and they have been successfully employed in biological experiments. However, the fabrication method using MPTS has potential disadvantages owing to its encapsulation mechanism. When MPTS attaches to the surface of QDs via ligand exchange, the crystal structure of the QDs may be damaged, causing a significant decrease in the quantum yield (QY) [22]. In the case of Ag or Au NPs labeled with Raman labeling compounds (RLCs), MPTS can replace the RLCs. Consequently, the number of RLCs reduces after silica encapsulation, and the signal intensity of the Raman scattering decreases significantly. To avoid these problems, it is necessary to develop a silica encapsulation method without thiol-containing silica precursors that directly attach to the surface of optical NPs.

Trimethoxy(2-phenylethyl)silane (TMPS) is a silane derivative containing one phenylethyl group and three methoxy groups attached to a silicon atom. Therefore, it can be used to introduce a silane group into composite materials because the phenylethyl group of TMPS can adhere to hydrophobic surfaces. Although TMPS has been used for the silica coating of QD NPs on silica templates, it has been limited to hydrophobic silica and QDs [23]. Owing to the high demand for silica coatings for composite structures with hydrophobic surfaces, it is necessary to develop general methods capable of coating non-hydrophilic surfaces with silica to expand their applicability.

This paper presents a generally applicable method for encapsulating optical NP-embedded silica NPs using TMPS. By substituting the reagent for silica encapsulation from MPTS to TMPS, which cannot interact directly with optical NPs, the ligand exchange or detachment of the RLCs is inhibited during the silica encapsulation step. Compared with previously reported silica-encapsulated optical NPs, such as silica-encapsulated multiple QD-embedded silica NPs (SiO_2_@QD@SiO_2_ NPs; QD^2^) or metal NPs with RLC-embedded silica particles (SiO_2_@Au_2-NT_@SiO_2_), the optical properties, such as the QY or SERS signal intensity, were only slightly reduced after silica encapsulation. Finally, the thickness of the silica shell can be controlled by adjusting the amount of TEOS used for additional silica shell formation.

## 2. Materials and Methods

### 2.1. Influence of Silica

For this study, tetraethylorthosilicate (TEOS), 3-mercaptopropyl trimethoxysilane (MPTS), chloroform, tetrakis(hydroxymethyl)-phosphonium chloride (THPC), polyvinylpyrrolidone (PVP, MW ~40,000), gold(III) chloride trihydrate (HAuCl_4_∙3H_2_O), ascorbic acid (AA), 3-aminopropyl triethoxysilane (APTES), 2-naphthalenethiol (2-NT), and trimethoxy(2-phenylethyl)silane (TMPS) were purchased from Sigma Aldrich (St. Louis, MO, USA). Dichloromethane and sodium hydroxide (NaOH) were purchased from Samchun (Pyeongtaek, Republic of Korea). Ethyl alcohol (EtOH, 99.9% purity) and aqueous ammonium hydroxide (NH_4_OH, 27% purity) were purchased from Daejung (Siheung, Republic of Korea). The quantum dots (QDs) were purchased from Zeus (Osan, Republic of Korea). These materials did not undergo any purification after purchase.

Transmission electron microscopy (TEM) images of the NPs were obtained using the JEM-2010 system (JEOL, Tokyo, Japan). The ultraviolet–visible (UV-Vis) light absorbance spectra of the NPs were obtained using an Optizen Pop UV/Vis spectrophotometer (Mecasys, Daejeon, Republic of Korea). The photoluminescence (PL) emission spectra of the NPs were obtained using a Cary Eclipse spectrophotometer (Agilent Technologies, Santa Clara, CA, USA). The quantum yield (QY) of NPs was measured using a QE-2000 instrument (Otsuka Electronics, Osaka, Japan). Raman spectra were obtained using a DXR Raman microscope (Thermo Fisher Scientific, Madison, WI, USA) equipped with a 780 nm diode laser. The samples prepared in solution were subjected to Raman measurements using capillary tubes. Each spectrum was measured in the range of 500–1800 cm^−1^ with a spectral resolution of 0.5 cm^−1^, and the SERS signals were collected with a backscattering geometry using a ×10 objective lens.

### 2.2. Preparation of Thiol- or Amine-Functionalized SiO_2_NPs (SiO_2-_SH or SiO_2-_NH_2_)

Approximately 150 nm of silica NPs (SiO_2_ NPs) were synthesized using the Stöber method with modification [24]. First, 40 mL of EtOH was mixed with 1 mL each of distilled water and 1.6 mL of TEOS. Subsequently, the mixture was mixed after adding 3 mL of NH_4_OH for 20 h at 25 °C. The SiO_2_ NPs were washed with EtOH several times by using centrifugation at 8500 rpm for 15 min and redispersed in EtOH.

To introduce the thiol groups onto the surface of SiO_2_ NPs, 10 μL of MPTS, 10 μL of distilled water, and 2.5 μL of NH_4_OH were added into 980 μL of SiO_2_ mixture in EtOH (1 mg/mL). The mixture was incubated for 1 h at 50 °C, and thiol-functionalized SiO_2_ NPs were washed with EtOH several times by using centrifugation at 8500 rpm for 15 min. After washing, the thiol-functionalized SiO_2_ NPs were redispersed in EtOH (10 mg/mL). To introduce amine groups on the surface of the SiO_2_ NPs, the same fabrication process was used, except that the MPTS was substituted with the same amount of APTES. The prepared 980 μL of SiO_2_ mixture (1 mg/mL) was stirred with 10 μL of APTES, 10 μL of distilled water, and 2.5 μL of NH_4_OH for 1 h at 50 °C. After the reaction, the amine-functionalized SiO_2_ NPs were washed with EtOH several times by using centrifugation at 8500 rpm for 15 min and redispersed in EtOH (10 mg/mL).

### 2.3. Preparation of SiO_2_@QD

The prepared SiO_2_-SH in EtOH (10 mg/mL, 1 mL) was mixed with 4 mL of dichloromethane and 6 mg of red QDs (100 mg/mL in toluene). The mixture was stirred using a rotator for 6 h at 50 rpm. To minimize particle aggregation, the reaction solution was sonicated for 1 min every 60 min. Subsequently, the SiO_2_@QDs mixture was washed using EtOH three times by using centrifugation at 7000 rpm for 10 min, and the washed particles were redispersed in EtOH after washing for storage.

### 2.4. Preparation of SiO_2_@QD@TMPS

Solvent exchange was performed to introduce TMPS to the surface of SiO_2_@QDs. The solvent of the SiO_2_@QD mixture was removed by using centrifugation at 7000 rpm for 10 min, and the SiO_2_@QD was dispersed in 20 mL of ethanol/chloroform (2:1, in terms of the volume ratio) mixture. To this mixture, 1.2 mL of distilled water and 0.8 mL of NH_4_OH were added while stirring. Subsequently, 140 μL of TEOS and 60 μL of TMPS were added to the mixture, and the mixture was stirred for 6 h at 25 °C. To reduce particle aggregation, the reaction mixture was sonicated every 1 h. The reaction mixture was washed several times with EtOH by using centrifugation at 7000 rpm for 10 min, and the washed particles were redispersed in 5 mL of EtOH.

### 2.5. Preparation of SiO_2_@QD@TMPS-SiO_2_

To encapsulate SiO_2_@QD@TMPS with a silica shell, 50 µL of D.W, 50 µL of TEOS, and 50 µL of NH_4_OH were added to the SiO_2_@QD@TMPS mixture and stirred using a rotator for 20 h at 25 °C. The final particles were washed by using centrifugation at 7000 rpm for 10 min and redispersed in EtOH.

For varying the thickness of the silica shell, the outer layer of SiO_2_@QD@TMPS, was accomplished by adjusting the amount of TEOS used. The silica encapsulation process of the outer layer of SiO_2_@QD@TMPS was conducted by adjusting the amount of TEOS to 25, 50, and 100 µL, respectively, while leaving the other reaction conditions unchanged. After the silica encapsulation process, the SiO_2_@QD@TMPS-SiO_2_ mixture was washed several times with EtOH by using centrifugation at 7000 rpm for 10 min, and the washed particles were dispersed in EtOH.

### 2.6. Preparation of SiO_2_@Au

SiO_2_@Au was fabricated based on the method detailed previously [25,26]. First, the 3–5 nm size of Au NPs were fabricated using the THPC method [27]. In detail, 1.5 mL of 0.2 M NaOH solution, 12 μL of THPC, and 1.5 mL of 50 mM HAuCl_4_∙3H_2_O solution were added sequentially into 47.5 mL of deionized water. The mixture was stirred for 1 h at room temperature, and after the reaction, the mixture was stored in a 4 °C refrigerator for at least two days prior to use. To attach Au NPs onto the silica template, 10 mL of Au NP mixture was mixed in 200 μL of prepared SiO_2_–NH_2_ (10 mg/mL). The mixture was incubated overnight at room temperature. Silica NPs with Au NPs embedded as a seed (SiO_2_@ seed Au NPs) were obtained after washing with EtOH several times by using centrifugation at 8000 rpm for 10 min and were redispersed in 2 mL of PVP solution (0.1%, *w*/*v*).

For the growth of Au NPs on SiO_2_@ seed Au NPs, 10 mg of PVP was dissolved in 9.8 mL of deionized water, and 200 μL of SiO_2_@seed Au NPs were mixed with this solution. To this solution, 20 μL of 10 mM HAuCl_4_∙3H_2_O solution and 40 μL of 10 mM ascorbic acid solution were added every 5 min, until the final concentration of Au^3+^ reached 500 μM. After the reaction, Au NP-embedded silica NPs (SiO_2_@Au NPs) were obtained by washing with EtOH several times by using centrifugation at 8000 rpm for 10 min and redispersed in EtOH to adjust the concentration of NPs to 0.1 mg/mL.

### 2.7. Preparation of 2-NT Labeled SiO_2_@Au NPs (SiO_2_@Au_2-NT_ NPs)

First, 1 mL of 2 mM 2-NT was prepared using an EtOH/DMF mixture (1:9 volume ratio) as the Raman labeling compound (RLC) solution. SiO_2_@Au mixture (1 mL) was transferred into a microtube, and the solvent of mixture was decanted after centrifugation at 8000 rpm for 10 min. The particles were redispersed in the prepared RLC solution, and the mixture was shaken for 1 h at room temperature. After the reaction, SiO_2_ @Au_2-NT_ NPs were obtained after washing with EtOH several times by using centrifugation at 8000 rpm for 10 min and then redispersed in 1 mL of EtOH to adjust the particle concentration at 0.1 mg/mL.

### 2.8. Silica Encapsulation of SiO_2_@Au_2-NT_ NPs (SiO_2_@Au_2-NT_@SiO_2_ NPs)

Silica encapsulation of SiO_2_@Au_2-NT_ NPs was conducted via a similar method with SiO_2_@QD NPs. The solvent of SiO_2_@Au_2-NT_ NP mixture (500 μL, 0.05 mg) was removed after centrifugation at 8000 rpm for 10 min, and SiO_2_ @Au_2-NT_ NPs were dispersed in 200 μL of EtOH/CHCl_3_ (2:1, in terms of the volume ratio) mixture. To this mixture, 12 μL of deionized water, 8 μL of NH_4_OH, 1.4 μL of TEOS, and 0.6 μL of TMPS were added sequentially. While incubating the mixture at room temperature, the mixture was sonicated every 1 h to prevent the aggregation of particles. The reaction was continued for 6 h, and TMPS-introduced SiO_2_@Au_2-NT_ NPs (SiO_2_@Au_2-NT_@TMPS NPs) were washed with EtOH several times by using centrifugation at 8000 rpm for 10 min. After washing, SiO_2_@Au_2-NT_@TMPS NPs were dispersed in 2.5 mL of EtOH, and 25 μL of deionized water and 25 μL of NH_4_OH were added to the mixture. To this mixture, 5 μL of TEOS was added every 30 min, five times in total. The reaction was kept for an additional 18 h at room temperature. SiO_2_@Au_2-NT_@SiO_2_ NPs were obtained after washing with EtOH several times by using centrifugation at 8000 rpm for 10 min and were redispersed in 1 mL of EtOH.

## 3. Results and Discussion

### 3.1. Fabrication of Silica-Encapsulated QD-Embedded Silica NPs with TMPS (SiO_2_@QD@TMPS-SiO_2_)

**Figure 1:**
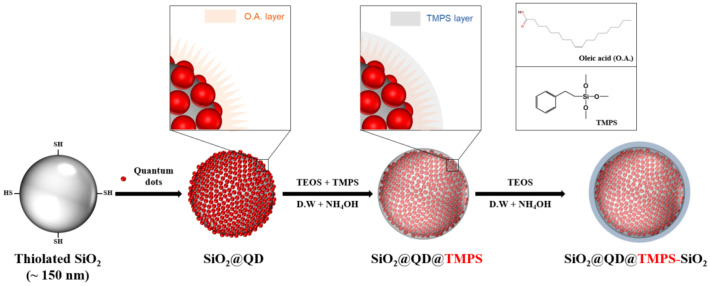
Synthesis of silica-encapsulated QD-embedded silica nanoparticles with TMPS (SiO_2_@QD@TMPS-SiO_2_).

**Figure 2:**
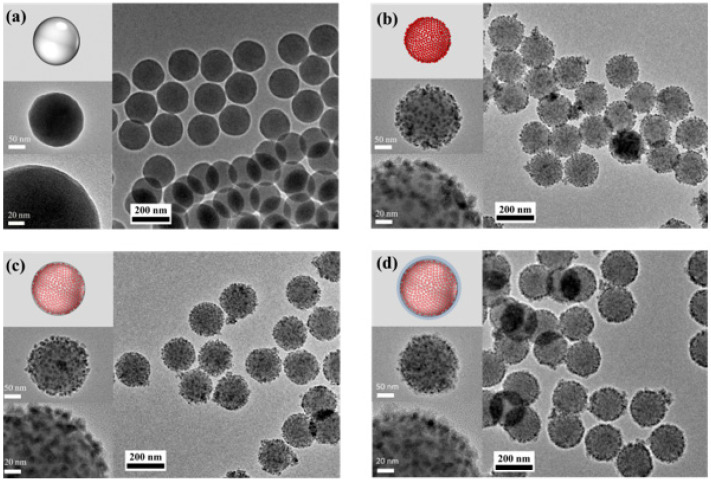
Illustrations and TEM images of each particle in the synthesis step: (**a**) SiO_2_ NPs, (**b**) SiO_2_@QD, (**c**) SiO_2_@QD@TMPS, and (**d**) SiO_2_@QD@TMPS-SiO_2_.

Figure 1 shows the fabrication process of the silica-encapsulated QD-embedded silica NP with TMPS (SiO_2_@QD@TMPS-SiO_2_). Compared to previous attempts used to fabricate silica–QD hybrid NPs via hydrophobic interactions by introducing octadecyl groups on their surfaces, silica NPs (SiO_2_ NPs) fabricated using the Stöber method were functionalized with thiol groups. Because thiol groups on the SiO_2_ NP surface do not interact with each other, SiO_2_ NPs do not undergo aggregation during the fabrication process, whereas the previous attempts have caused aggregation of silica NPs due to the hydrophobic interaction between the octadecyl groups. The average size of the SiO_2_ NPs was 175.0 ± 4.04 nm (Figure 2a and Figure S1). As shown in Figure 2b, the QDs were densely embedded onto the surface of the SiO_2_ NPs, in which thiol groups were introduced, via the ligand exchange method. To encapsulate the SiO_2_@QD with silica, TMPS was used as the silica precursor with a hydrophobic moiety. Although the 2-phenylethyl group was shorter than the octadecyl group and more difficult to interact with the oleyl group owing to the difference in the molecular structure, the hydrophobic interaction between the oleyl group and the 2-phenylethyl group was sufficiently strong to surround the SiO_2_@QDs with TMPS (Figure 2c). To increase the thickness of the silica shell at the outer layer of the SiO_2_@QD@TMPS, additional silica encapsulation was provided using tetraethyl orthosilicate (TEOS) as a silica source (Figure 2d). As shown in Figure 2b–d), there was no significant leaching of the QDs during the silica encapsulation process.

### 3.2. Optical Properties of SiO_2_@QD@TMPS-SiO_2_

**Figure 3:**
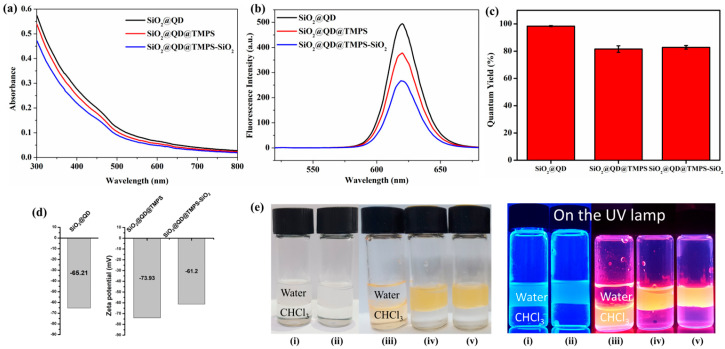
(**a**) UV−vis absorption spectra, (**b**) PL spectra, and (**c**) QYs for each step after the introduction of QDs during the synthesis of SiO_2_@QD@TMPS-SiO_2_. (**d**) Zeta potential of SiO_2_@QD, SiO_2_@QD@TMPS, and SiO_2_@QD@TMPS−SiO_2_. (**e**) Optical images under daylight (left) and UV light (right) of (i) SiO_2_ NPs, (ii) Thiolated SiO_2_ NPs, (iii) SiO_2_@QD, (iv) SiO_2_@QD@TMPS, and (v) SiO_2_@QD@TMPS−SiO_2_.

The optical properties of the fabricated NPs were evaluated. In principle, SiO_2_@QD, SiO_2_@QD@TMPS, and SiO_2_@QD@TMPS-SiO_2_ exhibited similar UV-Vis absorption and PL spectra (Figure 3a,b) which originated from the QDs. These results show that the optical properties of the NPs (particularly the QDs) did not change after the silica encapsulation process. The particles mostly absorbed UV light at short wavelengths (~300 to 400 nm), and the degree of absorbance decreased with increasing wavelength.

In terms of the PL spectra, the particles emitted light at a wavelength of 620 nm, which is the same as the light emitted from the individual QDs. Among the three types of NPs, SiO_2_@QDs exhibited the highest PL intensity (494.59 in arbitrary units). In contrast, SiO_2_@QD@TMPS-SiO_2_ exhibited the lowest PL intensity (290.05 in arbitrary units). This phenomenon can be attributed to QD damage during fabrication. In addition, this originates from the thickness of the silica shell. The light that arrived at or was emitted from the SiO_2_@QD did not undergo significant interference because the QDs of the SiO_2_@QD were exposed. Meanwhile, in the cases of SiO_2_@QD@TMPS and SiO_2_@QD@TMPS-SiO_2_ where the silica shell existed in the outer layer, the absorption or emission of light was interfered by the silica shell. Although the silica layer was relatively transparent, light scattering was inhibited by the silica shell.

To confirm the change in the QY after fabrication, the QYs of SiO_2_@QD, SiO_2_@QD@TMPS, and SiO_2_@QD@TMPS-SiO_2_ were also measured (Figure 3c). In the cases of SiO_2_@QD and SiO_2_@QD@TMPS, the QYs were similar (47.5% and 49.4%, respectively). Meanwhile, the QY decreased slightly after additional silica encapsulation with TEOS (49.4% and 41.3% for SiO_2_@QD@TMPS and SiO_2_@QD@TMPS-SiO_2_, respectively). This was likely due to the interference of the outermost silica layer, as in the case of the PL intensity. Although the QY was reduced after additional silica encapsulation, the QY and PL intensity of SiO_2_@QD@TMPS-SiO_2_ were relatively good; in particular, the QY of SiO_2_@QD@TMPS-SiO_2_ was higher than that of QD^2^, which was fabricated using the previous method [19]. The NPs in all phases were well dispersed, which is supported by the zeta potential data. As shown in Figure 3d, the high negative zeta potential values indicates that the NPs in all phases had good dispersion stability and were not aggregated [28].

### 3.3. Hydrophilicity of SiO_2_@QD@TMPS-SiO_2_

To confirm the silica encapsulation indirectly, hydrophilic properties of the NPs were evaluated at each fabrication step under daylight and UV light (Figure 3e). First, each mixtures containing SiO_2_ NPs and thiolated SiO_2_ NPs were well dispersed in the water phase and were not observed in the hydrophobic chloroform layer. These phenomena occurred owing to the functional groups which located onto the surface of each particle; silanol groups (Si-OH) on SiO_2_ NPs and thiol groups (Si-SH) on thiolated SiO_2_ NPs. Because these groups can be deprotonated in mild conditions, negative charges which enhance the colloidal stability and hydrophilicity of NPs could be existed onto the surface of NPs. When the QDs coated with oleic acid were attached to the surface of the thiolated SiO_2_ NPs, SiO_2_@QDs exhibited strong hydrophobicity owing to the oleyl group of the QD ligands and were transferred into the organic phase. In contrast, SiO_2_@QD@TMPS and SiO_2_@QD@TMPS-SiO_2_ were well dispersed in the water phase. These results indicate that the outer functional groups were substituted from hydrophobic oleic acid to another hydrophilic functional groups. As mentioned at Section 3.1, the 2-phenylethyl group of TMPS, which has hydrophobic property was intercalated between oleic acid via hydrophobic interactions. Therefore, the exposed functional groups that were present onto the surface of SiO_2_@QD@TMPS and SiO_2_@QD@TMPS-SiO_2_ should be the silanol group which originated from the hydrolysis of methoxy group. The dispersity of NPs which QDs were embedded (SiO_2_@QD, SiO_2_@QD@TMPS, and SiO_2_@QD@TMPS-SiO_2_) also could be confirmed by observing luminescence from the QDs under UV light irradiation.

### 3.4. Control of Silica Shell Thickness of SiO_2_@QD@TMPS-SiO_2_

**Figure 4:**
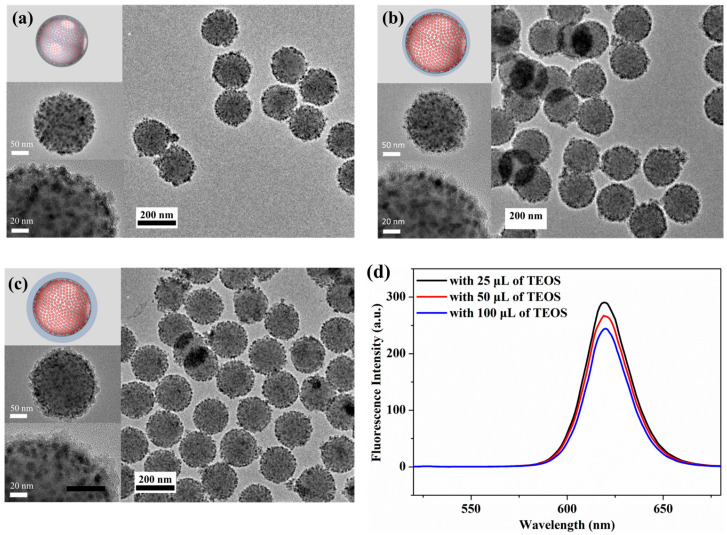
Illustration and TEM images of fabricated SiO_2_@QD@TMPS-SiO_2_ with different amounts of TEOS: (**a**) 25 μL (**b**) 50 μL, and (**c**) 100 μL. (**d**) PL spectra of fabricated nanoparticles.

According to the applications of NPs with optical properties, controlling of the thickness of outer silica shell should be necessary. With this reason, we tried to control the thickness of fabricated SiO_2_@QD@TMPS-SiO_2_ by adjusting the amount of TEOS used in the final silica encapsulation step. As shown in Figure 4a–c, the thickness of the silica shell layer increases with the increase in the amount of TEOS (25, 50, and 100 μL, respectively). Although the thickness of the silica layer varied, the morphology and other aspects of SiO_2_@QD@TMPS-SiO_2_ did not significantly differ, particularly in terms of the attachment of the QDs. Furthermore, varying the thickness of the silica layer did not significantly change the high negative zeta potential values of the NPs, which were presumed to maintain good dispersion (Figure S2).

The influence of the silica shell thickness on the optical properties of SiO_2_@QD@TMPS-SiO_2_ was evaluated. Regardless of the silica shell thickness, the PL spectra of SiO_2_@QD@TMPS-SiO_2_ were similar except for the maximum intensity (Figure 4d). The maximum fluorescence intensity slightly decreased with an increasing of the silica shell thickness (from 290.05 to 244.25, in arbitrary units). As in the case of PL intensity, the QY of each particle decreased with increasing silica shell thickness (Figure S3, 42.6%, 41.3%, and 36.2%, respectively). These results may be related to the thickness of the outer silica shell layer. Even though the silica shell layer was almost transparent, the crossing of light, which came from the light source for excitation or fluorescence from QDs as an emission, could be inhibited via scattering or weak absorption. However, although the PL intensity and QY decreased in SiO_2_@QD@TMPS-SiO_2_ with a thick silica shell compared to the thin silica shell, SiO_2_@QD@TMPS-SiO_2_ with a thick silica shell can be used for other applications because it still has a relatively high PL intensity and QY, as compared to other QD–silica hybrid NPs.

### 3.5. Fabrication of Silica-Encapsulated Gold NP-Embedded Silica NPs with TMPS (SiO_2_@Au_2-NT_@SiO_2_)

**Figure 5:**
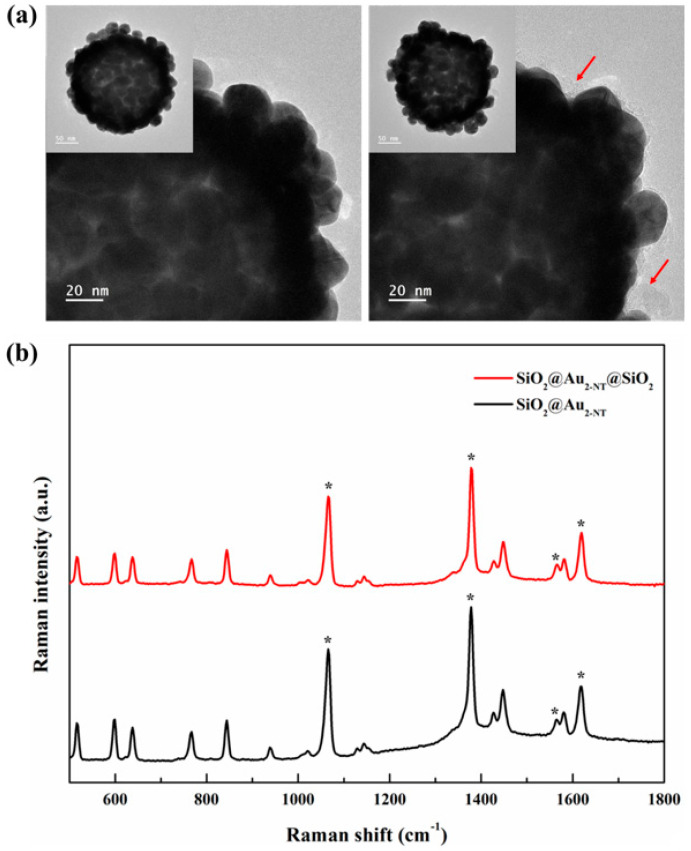
TEM images of (**a**) SiO_2_@Au_2-NT_ (left) and SiO_2_@Au_2-NT_@SiO_2_ (right). The red arrow indicates the silica shell. (**b**) Raman spectra of SiO_2_@Au_2-NT_ and SiO_2_@Au_2-NT_@SiO_2_. The “*” indicates the representative peaks of 2-NT.

The silica encapsulation method using TMPS has also been adopted for noble metal NP-embedded silica NPs. Gold NP-assembled silica NPs (SiO_2_@Au), which have shown excellent performance as near-infrared SERS nanoprobes, were selected as the target for silica encapsulation to ensure the feasibility of further surface modification [25]. As Raman label compound (RLC), 2-naphthalenethiol (2-NT), of which a single thiol group was conjugated at 2-position of naphthalene, was selected due to its strong hydrophobicity compared with another commonly used RLCs such as 4-mercaptobenzoic acid (4-MBA) or 4-aminothiophenol (4-ATP). To fabricate SiO_2_@Au_2-NT_, 2-NT was labeled onto the surface of SiO_2_@Au. Because 2-NT was attached to the surface of SiO_2_@Au via an interaction between thiol and Au, the outer-most functional group of SiO_2_@Au_2-NT_ was the naphthyl group, which represents strong hydrophobicity. After labeling, SiO_2_@Au_2-NT_ were encapsulated with a silica shell using TMPS and TEOS as silica precursors such as SiO_2_@QD@TMPS. As in the case of oleyl groups of QDs, the naphthyl group interacted with the 2-phenylethyl group. However, differing from the oleyl groups, which can interact with the 2-phenylethyl group via hydrophobic interaction, naphthyl groups can interact with the 2-phenylethyl group via pi–pi interactions, which are stronger than simple hydrophobic interactions. After silica encapsulation using TMPS and TEOS, the silica shell layer was grown by using TEOS once more to fabricate SiO_2_@Au_2-NT_@SiO_2_. As shown in Figure 5a, the overall morphology of the NPs does not change significantly, unlike silica encapsulation with TMPS. Notably, a thin silica shell layer can be observed in the outer layer of SiO_2_@Au_2-NT_@SiO_2_ in the TEM image. These results indicate SiO_2_@Au_2-NT_@SiO_2_ was successfully fabricated using TMPS.

### 3.6. Influence of Silica Encapsulation on the Optical Properties of SiO_2_@Au_2-NT_

To determine the influence of silica encapsulation with TMPS to the Raman signal intensity of the fabricated NPs, the Raman spectra of SiO_2_@Au_2-NT_ were acquired before and after silica encapsulation (SiO_2_@Au_2-NT_ and SiO_2_@Au_2-NT_@SiO_2_, respectively). As shown in Figure 5b, the spectral patterns of SiO_2_@Au_2-NT_ and SiO_2_@Au_2-NT_@SiO_2_ were largely the same, and representative peaks were observed at 1065, 1378, 1565, and 1619 cm^−1^ for both these particles. In particular, the peaks at 1565 and 1619 cm^−1^ are assigned to the aromatic ring stretching mode of the 2-NT unit, which means that 2-NT exists on the surface of SiO_2_@Au regardless of the TMPS treatment [29,30]. Moreover, the Raman signal intensities of SiO_2_@Au_2-NT_ and SiO_2_@Au_2-NT_@SiO_2_ at 1619 cm^−1^ were similar (5204 vs. 4146, in arbitrary units) at the same particle concentration. These results indicate that the silica encapsulation of SiO_2_@Au_2-NT_ with TMPS did not influence the optical properties, specifically the Raman signal intensity.

## 4. Conclusions

We introduced a novel generalizable method for the encapsulation of NP-embedded silica NPs with TMPS and fabricated SiO_2_@QD@TMPS-SiO_2_ NPs and SiO_2_@Au_2-NT_@SiO_2_ NPs using the introduced method. In the case of the SiO_2_@QD@TMPS-SiO_2_ NPs, TMPS interacted with hydrophobic oleic acid that was attached to the surface of the QD and formed a silica shell layer without ligand exchange. The PL and QY of SiO_2_@QD@TMPS-SiO_2_ NPs were not significantly lower than those of SiO_2_@QD. The thickness of the outer silica shell was controlled by adjusting the amount of TEOS used during the silica shell formation step, which was the last step in the fabrication. Silica encapsulation with TMPS was also applied to SiO_2_@Au_2-NT_ NPs, and the outer silica shell was formed successfully without any significant change of morphology of NPs. Raman signal spectra of the NPs also did not change significantly during silica encapsulation. Based on these results, we expect that the proposed silica encapsulation method using TMPS can be applied to not only other hydrophobic RLC-labeled SiO_2_@Au NPs, but also to NPs with hydrophobic surfaces.

## Data Availability

All data generated or analyzed during this study are included in this manuscript and its Supplementary Materials.

## Supplementary Materials

The following supporting information can be downloaded at: https://www.mdpi.com/article/10.3390/nano13142145/s1, Figure S1: Histogram of particle size distribution of SiO_2_ NPs, Figure S2: Zeta potential of SiO_2_@QD@TMPS-SiO_2_ with different amount of TEOS, Figure S3: Quantum yield of fabricated SiO_2_@QD@TMPS-SiO_2_ with different amount of TEOS.
