# Supplementary material for: Silica Encapsulation of Hydrophobic Optical NP-Embedded Silica Particles with Trimethoxy(2-Phenylethyl)silane
Source: Nanomaterials (Basel). 2023 Jul 24;13(14):2145. doi: 10.3390/nano13142145 (PMC10384416; doi:10.3390/nano13142145)
Supplement: Supplementary file 1 [file nanomaterials-13-02145-s001.zip › nanomaterials-2339925-supplementary.pdf]

nanomaterials

Supporting Information

Silica Encapsulation of Hydrophobic Optical NP-Embedded Silica Particles with Trimethoxy(2-Phenylethyl)silane

Eunil Hahm [1], Ahla Jo [1], Eun Ji Kang [1], Kwanghee Yoo [1], Min-Sup Shin [1], Jaehyun An [1], Xuan-Hung Pham [1], Hyung-Mo Kim [1], Homan Kang [2], Jaehi Kim [1,*], and Bong-Hyun Jun [1,*]

[1] Department of Bioscience and Biotechnology, Konkuk University, Seoul 05029, Republic of Korea; greenice@konkuk.ac.kr (E.H.); iamara0421@konkuk.ac.kr (A.J.); ejkang@konkuk.ac.kr (E.J.K.); heu1997@konkuk.ac.kr (K.Y.); dnjzj159159@konkuk.ac.kr (M.-S.S.); wogus4067@naver.com (J.A.); phamricky@gmail.com (X.-H.P.); hmkim0109@konkuk.ac.kr (H.-M.K.)

[2] Gordon Center for Medical Imaging, Department of Radiology, Massachusetts General Hospital, Harvard Medical School, Boston, MA 02114, USA; hkang7@mgh.harvard.edu

* Correspondence: susia45@gmail.com (J.K.); bjun@konkuk.ac.kr (B.-H.J.)

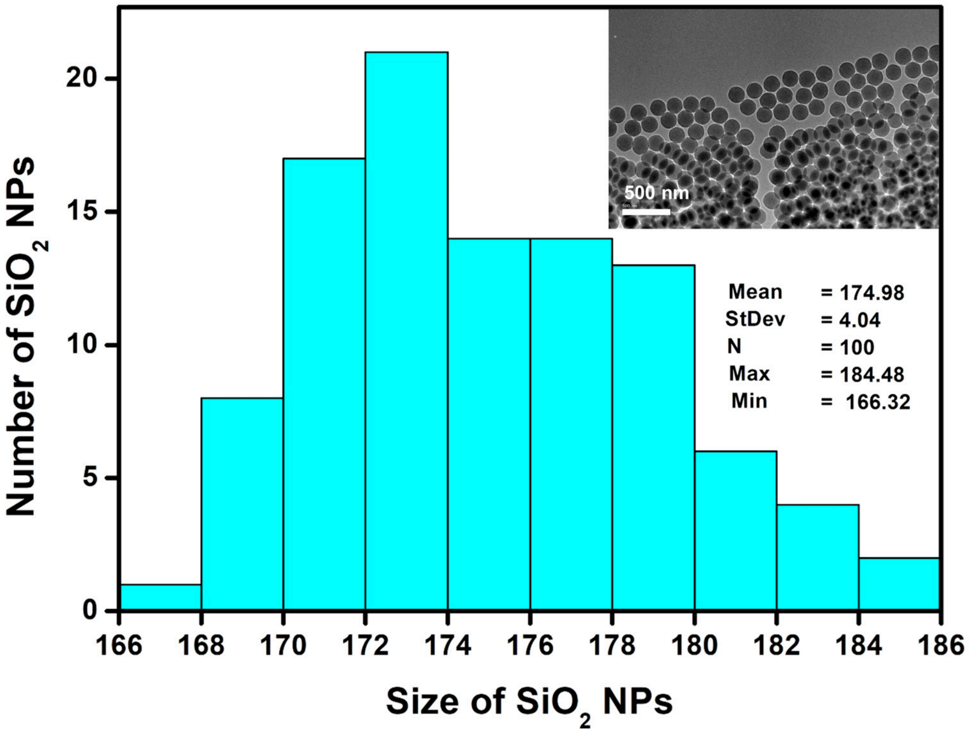

Figure S1. Histogram of particle size distribution of SiO_2 NPs (inset. TEM image of SiO_2 NPs).

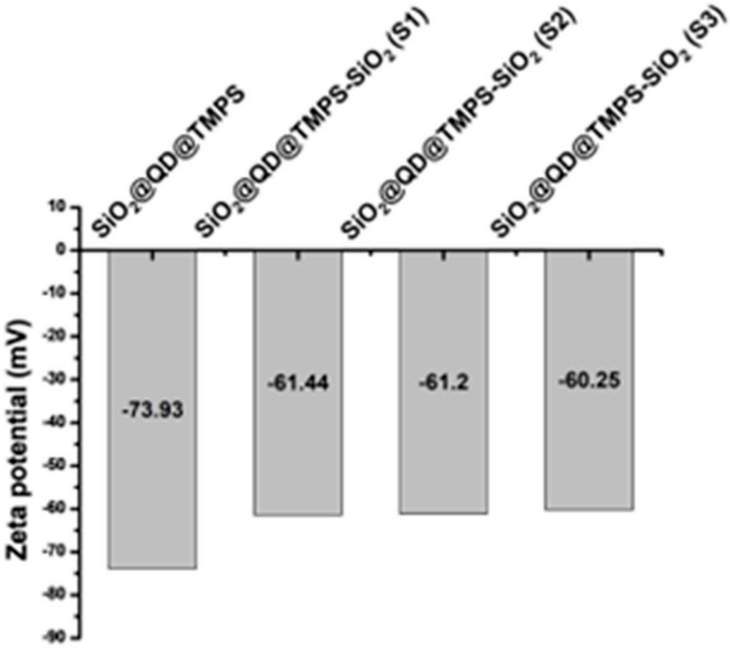

Figure S2. Zeta potential of SiO_2@QD@TMPS-SiO_2 with different amount of TEOS.

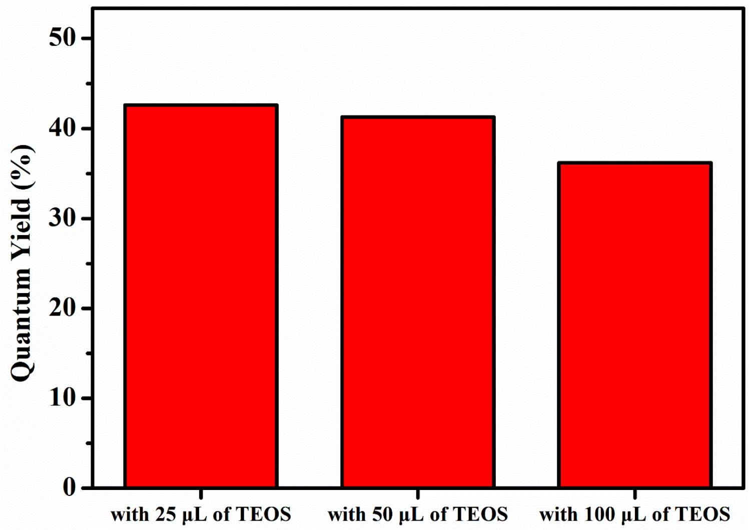

Figure S3. Quantum yield of fabricated SiO_2@QD@TMPS-SiO_2 with different amount of TEOS.
